# Ufasomes Mediated Cutaneous Delivery of Dexamethasone: Formulation and Evaluation of Anti-Inflammatory Activity by Carrageenin-Induced Rat Paw Edema Model

**DOI:** 10.1155/2013/680580

**Published:** 2012-11-25

**Authors:** Rajkamal Mittal, Arvind Sharma, Sandeep Arora

**Affiliations:** Chitkara College of Pharmacy, Chitkara University, Punjab 140401, India

## Abstract

The purpose of study is to formulate and evaluate ufasomal gel of dexamethasone. Ufasomal suspension was made by sonication method using different concentrations of Span 80, Span 20 and cholesterol along with 25 mg of drug. Ufasomal gel was formulated by hydration method using carbopol 940. Ufasomal vesicles appeared as spherical and multilamellar under Transmission Electron Microscope. Ufasomal formulation prepared with drug to oleic acid molar ratio 8:2 (UF-2) produced greater number of vesicles and greater entrapment efficiency. UF-2 was optimized for further evaluation. The transdermal permeation and skin partitioning of from optimized formulation was significantly higher (*P* < 0.05) as compared to plain drug and plain gel formulation which is due to presence of surfactant acting as permeation enhancer. Permeation of optimized formulation was found to be about 4.7 times higher than plain drug gel. Anti-inflammatory activity evaluated by inhibition Carrageenan induced rat paw edema model. Significant reduction of edema (*P* < 0.10) was observed in comparison to the commercial product. Hence oleic acid based vesicles can be used as alternate carrier for topical delivery.

## 1. Introduction

Dexamethasone is a glucocorticoid with a relevant clinical use mainly due to its anti-inflammatory and immunosuppressive effects. However, the great number of side effects, such as hypertension, hydroelectrolytic disorders, hyperglycemia, peptic ulcers, and glucosuria, restricts the use of dexamethasone in prolonged therapy [1]. Topical administration of dexamethasone is clinically used for the treatment of many ocular disorders, or diseases, like uveitis, [2] allergic conjunctivitis, [3] and corneal postoperative period, [4] as well as for the treatment of skin disorders such as atopic dermatitis, [5, 6] allergic dermatitis, eczematous dermatitis, [6, 7] psoriasis, acne rosacea, [8] and phimosis [9]. Over the last years many efforts have been made not only to improve the efficacy and bioavailability of drugs but also to reduce their adverse effects by means of the development of novel drug carrier systems [10].

In the past few decades, considerable attention has been focused on the development of new drug delivery system (NDDS). When the new drug or existing drug is given by altering the formulation and administered through different route, this process is called the novel drug delivery system. The NDDS should ideally fulfill two prerequisites. Firstly, it should deliver the drug at a rate directed by the needs of the body, over the period of treatment. Secondly, it should channel the active entity to the site of action. Conventional dosage forms including prolonged release dosage forms are unable to meet none of these. At present, no available drug delivery system behaves ideally, but sincere attempts have been made to achieve them through various novel approaches in drug delivery [11]. An increasing number of drugs are being added to the list of therapeutic agents that can be delivered into systemic circulation, in clinically effective concentrations, via the skin portal [12].

It has been documented and reported that unsaturated fatty acids such as oleic acid and linoleic acid have a tendency to form vesicles in the aqueous environment [13]. After about a decade of research it was conferred that saturated fatty acids with carbon atoms in the range of 8–12 undergo self-assemblage into vesicles in a pH-dependent manner [14]. Fatty acids being highly soluble tend to partition into artificial as well as natural membranes quite rapidly [15]. It has also been documented that fatty acid vesicles enhance the absorption of therapeutic molecules through the GIT, probably by forming mixed micelles or through chylomicron(s), thus enhancing the bioavailability of the bioactives [16]. It is well established fact that free fatty acids act as penetration enhancers for the bioactives through the stratum corneum [17]. Skin permeation potential of fatty acids varies with the chain length and branching. The penetration enhancement effect of fatty acid bears direct relation with the chain length; however direct relationship correlates up to carbon number 18, that is, C_18_. The skin permeation property of unsaturated fatty acids is higher than the corresponding saturated fatty acid. Further, fatty acid(s) containing Cis double bond exhibited higher penetration potential as compared to Trans form [18]. Skin irritation characteristics of fatty acid limit their use as penetration enhancer. The problem of skin irritation, however, could be addressed by using fatty acid vesicles as drug bearing carriers such as ufasomes. It has been shown that bilayer membrane possesses a fusogenic tendency due to its capability to lower the phase transition temperature of the lipids in the biological membrane. The vesicular membrane fuses with skin lipid bilayers, releasing its contents. Thus, it is hypothesized that fatty acid vesicles will act as a suitable carrier to enhance the penetration of bioactive agents through the stratum corneum with reduced toxicity. Moreover, fatty acid vesicles seem advantageous as they are easy to prepare as well as cost effective [19]. The present study involves the use of oleic acid vesicles to encapsulate dexamethasone and evaluates, its potential as an alternative drug delivery system for effective topical application.

## 2. Materials and Methods

Dexamethasone was obtained as a gift sample from IPZAH Pharmaceuticals Pvt. Ltd. (Patiala, India). Oleic acid was purchased from CDH (New Delhi, India). Sephadex G-50 and Dialysis membrane were purchased from Himedia (Mumbai, India). Carbopol 940 was purchased from Himedia (Mumbai, India). All other solvents used were of analytical grade, unless otherwise mentioned, and purchased from CDH.

### 2.1. Preparation of Fatty Acid Vesicles

Oleic acid vesicles were formulated by film hydration method, as reported earlier by [16] with slight modification. Different batches of ufasomes were formulated using different ratios of oleic acid, drug, and surfactant. Briefly, in a clean, dry, round bottom flask, the accurately weighed oleic acid of strength 80 mM, Span 20, and dexamethasone were dissolved in methanol followed by solvent evaporation under vacuum using a rotary evaporator (Perfit equipments, Ambala, India) under reduced pressure at 40°C to remove even the last traces of organic solvent. A dried film is formed in rotary evaporator and was left overnight for the removal of any possible traces of methanol and also to prevent the formation of emulsion due to the residual organic solvent. The dried film was then hydrated with PBS (pH 7.4) at ambient temperature for 1 h followed by sonication to form the uniform size vesicular dispersion. Optimization was performed by varying the ratios of oleic acid and dexamethasone (Table 1). Unentrapped drug was estimated by using dialysis method (Molecular weight cut of 12,000–14,000, Himedia, Ltd.). Briefly, 1% w/v of Carbopol 940 was dispersed into purified water with the help of a vortex shaker (Tarsons, Kolkata, India) and allowed to hydrate for 4-5 h. The pH value of the gel was adjusted to 7.4 using triethanolamine. During preparation of the gel, the solution was agitated slowly to obviate any air entrapment. Plain drug gel was prepared by using an equivalent amount of dexamethasone solution into the previously made Carbopol gel in a 2 : 1 ratio under gentle mechanical mixing for 5 min.

### 2.2. *In Vitro* Vesicle Characterization

Ufasomal formulations were characterized for different parameters like drug entrapment efficiency, vesicle shape, vesicle size, and size distribution, turbidity, zeta potential, and permeation across cellophane membrane and rat skin.

#### 2.2.1. Vesicle Shape

Fatty acid vesicles were visualized by using Moragagni 268D TEM with an accelerating voltage of 100 kV. A drop of the optimized formulation was placed onto a carbon-coated copper grid and it was negatively stained with 1% phosphotungstic acid (PTA). The grid was allowed to air-dry thoroughly and the samples were viewed on a transmission electron microscope [20].

**Figure 1:**
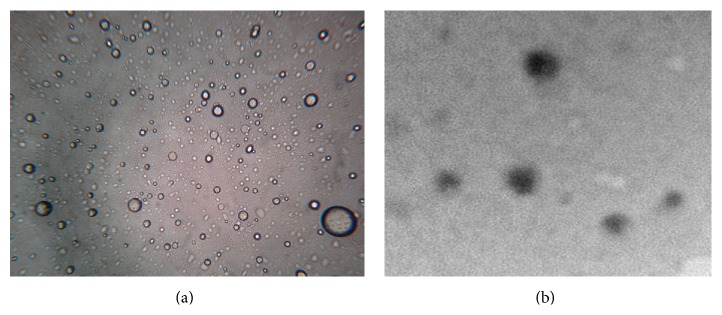
Vesicle morphology of optimized formulation (a) optical microscopy (400x magnification), (b) transmisson electron microscopy (TEM).

Vesicles without sonication were also visualized by using an optical microscope. A thin film of UF was spread on a slide and after placing cover slip it was observed under the optical microscope (OLYMPUS CH20i BIMF, 8F03730) (Figures 1(a) and 1(b)).

#### 2.2.2. Particle Size and Particle Size Distribution

**Figure 2:**
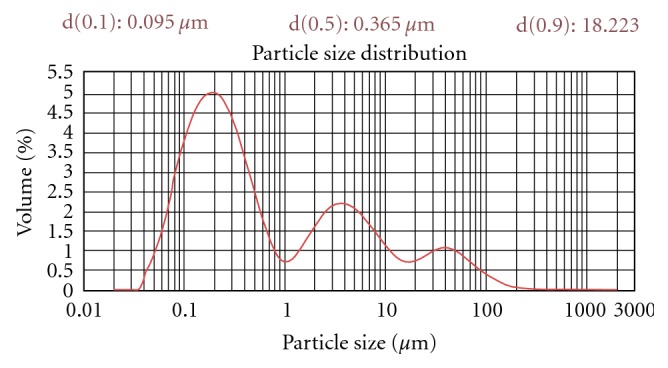
Dynamic light scattering (DLS) analysis of UF-2 formulation.

The size and size distribution of vesicles with different composition shown in Table 1 were determined by optical microscopy using stage eyepiece micrometer calibrated using micrometer scale. After sonication the vesicle size was determined by dynamic light scattering method (DLS), using a computerized inspection system (Malvern Zetamaster, ZEM 5002, Malvern, UK) and Figure 2 shows vesicle size distribution of optimized formulation. For vesicles size measurement the vesicular preparation was mixed with appropriate medium (for ufasomal formulation with 7% v/v ethanol) and filtered through 0.2 *μ*m polycarbonate membrane to minimize interference from particular matter. The measurements were conducted in triplicate in a multimodal mode of 200 and each at a medium stable count rate.

#### 2.2.3. Determination of Entrapment Efficiency

Entrapment Efficiency of dexamethasone from the UF-1, UF-2, and UF-3 formulations were estimated using dialysis method (Molecular weight cut of 12,000–14,000, HI Media, Ltd.). Optimized formulation was dissolved in PBS (pH 7.4) at a concentration of 1 mg/mL (the same concentration of dexamethasone as 1 mg/mL pure drug solution). This solution (2 mL in volume) was transferred to a dialysis bag (size cut off = 2.5 nm) immediately. The dialysis bag was placed in a 50 mL-beaker containing 40 mL PBS (pH 7.4). The outer phase was stirred continuously. At predetermined time intervals sample was withdrawn and replenished with same amount of receptor fluid. The absorbance of the outer phase was monitored at 241 nm using a spectrophotometer (Systronics Electronic Limited, Ahmedabad, India, AU-2701) in order to characterize the concentration of dexamethasone. Entrapment efficiency was expressed as percentage of total drug entrapped. The entrapment efficiency of prepared ufasomes was determined by subtracting the unentrapped drug from the total amount of drug used for the preparation of ufasomes. One has

(1) $$\begin{array}{c} \text{Percentage}\;\text{entrapment}=\;\frac{\text{Entrapped}\;\text{drug}\;\left(\mu \text{g}\right)}{\text{Total}\;\text{drug}\;\text{added}\;\left(\mu \text{g}\right)}\;\times 100. \end{array}$$

The entrapment efficiency calculated for various molar ratios is listed in Table 1.

#### 2.2.4. Turbidity Zeta Potential Measurements

Oleic acid vesicles were diluted with distilled water to give a total lipid concentration of 0.312 mM. After rapid mixing by sonication for 5 minutes the turbidity was measured as absorbance at 241 nm with an UV-visible spectrophotometer. The zeta potential of the all ufasomal formulations was determined in a Malvern Zetasizer using reagent blank. For zeta potential measurement vesicular suspension was mixed with the appropriate medium (for ufasomes with 7% v/v ethanol) and measurements were conducted in triplicate [21] (Table 1).

### 2.3. *In Vitro* Drug Release Using Cellophane Membrane

**Figure 3:**
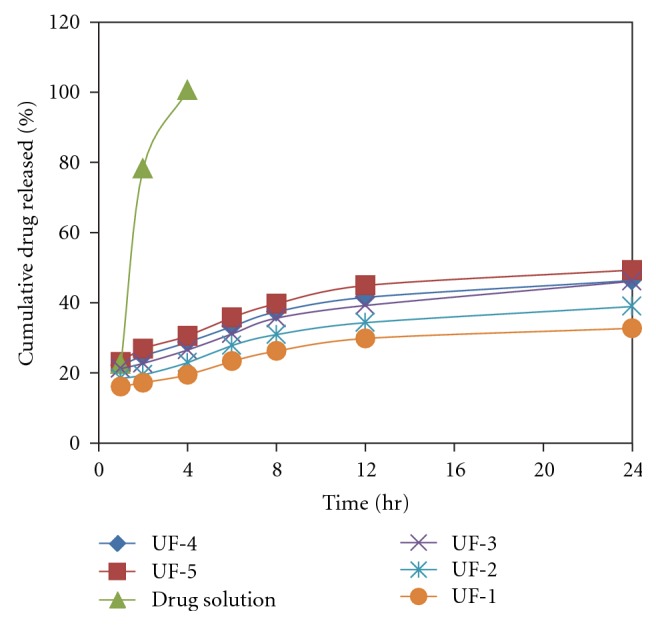
*In vitro* drug release profile of oleic acid vesicles.

*In vitro* release behavior of dexamethasone from vesicular formulations containing oleic acid and Span 20 was investigated using locally fabricated Franz glass diffusion cell and through the cellophane membrane (Molecular weight cut of 12,000–14,000, HI Media, Ltd.). Pure dexamethasone was dissolved in PBS (pH 7.4.) at a concentration of 1 mg/mL and used as control. The prepared complex was dissolved in PBS (pH 7.4) at a concentration of 1 mg/mL (the same concentration of dexamethasone as 1 mg/mL pure drug solution). This solution (2 mL in volume) was transferred to a dialysis bag (size cut off = 2.5 mm) immediately. The dialysis bag was placed in a 50 mL-beaker containing 40 mL PBS (pH 7.4). The outer phase was stirred continuously. At predetermined time intervals sample was withdrawn and replenished with same amount of receptor fluid. The absorbance of the outer phase was monitored at 241 nm spectrophotometrically in order to characterize the concentration of dexamethasone (Figure 3).

### 2.4. pH-Dependent Stability

The effect of pH on the stability and on the drug release behavior was monitored by incubating optimized vesicular dispersion with buffers of pH 8.5, 7.4, 6.5, and 5.5. At predetermined time intervals the samples were withdrawn and centrifuged at 14,000 rpm for 30 min [22]. The supernatant was analyzed for released free drug. The amount of drug leached was then calculated by the following formula:

(2) $$\begin{array}{c} \%\text{Drug}\;\text{diffused}=\frac{\text{Amount}\;\text{of}\;\text{free}\;\text{drug}}{\text{Total}\;\text{drug}}\times 100. \end{array}$$

**Figure 4:**
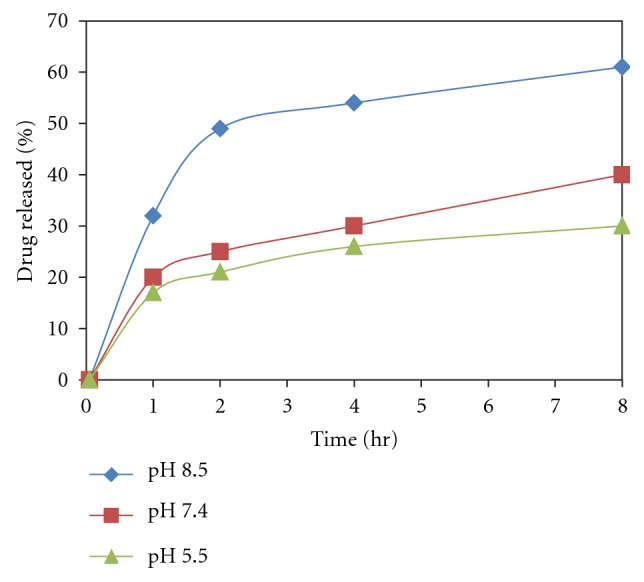
pH-dependent release behavior of drug from oleic acid vesicles dispersion.

**Figure 5:**
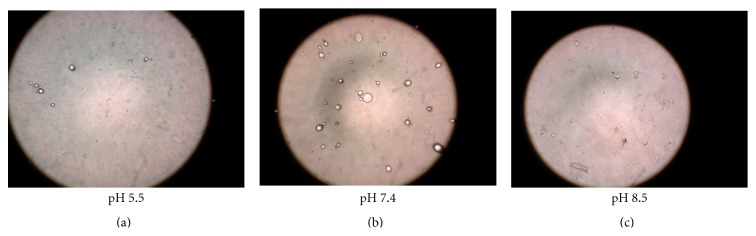
Photomicrograph of oleic acid vesicular dispersion incubated at different pH (400x magnification).

Simultaneously, the incubated vesicles were observed for any change in morphology and size using an optical microscope. The studies were performed in triplicate (Figures 4 and 5).

### 2.5. *In Vitro* Drug Release Using Locally Fabricated Franz Diffusion Cell

#### 2.5.1. Oleic Acid Vesicle Dispersion

The oleic acid vesicle dispersion was prepared as described previously. As the pH of skin is 7.4 so as to make the formulation compatible with skin, the pH of the dispersion was adjusted. The unentrapped drug was separated from the formulation by dialysis method (Molecular weight cut of 12,000–14,000, Himedia, Ltd.).

#### 2.5.2. Preparation of Plain Dexamethasone Gel

Dexamethasone containing Carbopol gel was prepared by the method reported by [23]. Briefly, 1% w/v of Carbopol 940 was dispersed into purified water with the help of a vortex shaker (Tarsons, Kolkata, India) and allowed to hydrate for 4-5 h. The pH value of the gel was adjusted to 7.4 using triethanolamine. During preparation of the gel, the solution was agitated slowly to avoid any air entrapment. Plain drug gel was prepared by using an equivalent amount of dexamethasone solution into the previously made Carbopol gel in a 2 : 1 ratio under gentle mechanical mixing for 5 min.

#### 2.5.3. Preparation of Rat Skin

Albino rat 5-6 weeks old weighing 100–120 g was sacrificed by chloroform inhalation. The hair of test animals was carefully trimmed short (<2 mm) with a pair of scissors and the abdominal skin was separated from the underlying connective tissue using scalpel. The excised skin was placed on aluminium foil and the dermal side of the skin was gently teased off for any adhering fat and/or subcutaneous tissue. The skin was then carefully checked through a magnifying glass to ensure that samples were free from any surface irregularities such as tiny holes or cervices in the portion that was used for transdermal permeation studies. The skin was washed with physiological buffer saline (pH 7.4) and freshly obtained skin was used in all experiments.

#### 2.5.4. Skin Permeation Studies

The *In vitro* skin permeation of dexamethasone from different formulations was studied using locally fabricated diffusion cell. The effective permeation area of the diffusion cell was 2.303 cm^2^. The receptor compartment contains 22.5 mL PBS (pH 7.4). Albino abdomen rat skin was mounted between the donor and receptor compartments. The donor compartment was maintained at 37 ± 1°C with constant stirring at 125 rpm. The UF-2 (2 mL) was applied to the epidermal surface of the rat skin. Samples were withdrawn through the sampling port of the diffusion cell at predetermined time intervals over 24 hr and analyzed. The receptor phase was immediately replenished with an equal volume of fresh buffer. The *in vitro* drug release study of ufasomal formulation was repeated with a cellophane membrane by using the same method as described above. Experiments were conducted to optimize the amount of dexamethasone that can be incorporated into the vesicles and to optimize the UF-2 formulation.

*Calculation of Permeation Parameters.* The cumulative amount of drug permeated per unit area was plotted as a function of time, and the steady state permeation rate (Jss) and lag time (LT, h) were calculated from the slope and X-intercept of the linear portion, respectively. The permeability coefficient (Ps, cm/hr) and other parameters were calculated from the following equation:

(3) $$\begin{array}{c} \text{LT}=\frac{H^{2}}{6D},\\ \text{Jss}=\text{Ps}\cdot \text{Cd}, \end{array}$$

where *H* = thickness of rat skin, Cd = amount of drug in donor compartment.

The enhancement ratio (ER) was calculated from following equation:

(4) $$\begin{array}{c} \text{ER}=\frac{\text{Transdermal}\;\text{flux}\;\text{from}\;\text{vesicular}\;\text{formulation}}{\text{Transdermal}\;\text{flux}\;\text{from}\;\text{plain}\;\text{drug}}. \end{array}$$

**Figure 6:**
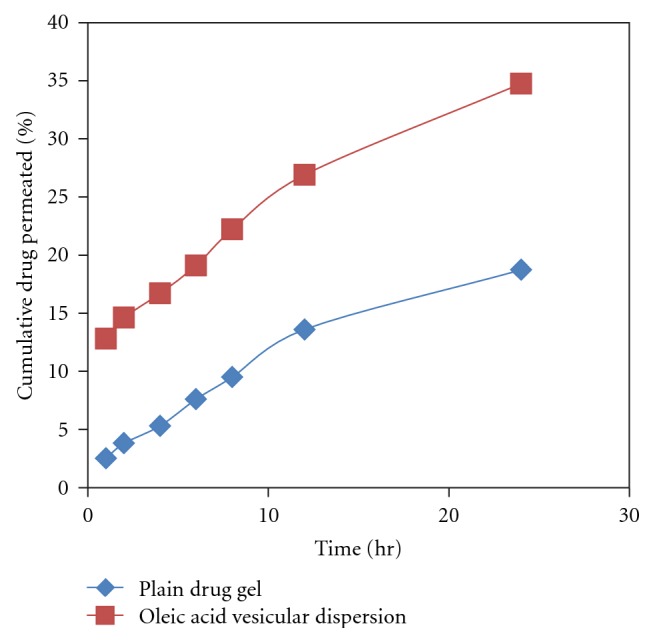
% Cumulative drug permeation through rat skin of plain drug gel and optimized formulation.

*Determination of Amount of Drug Deposited into the Skin.* In this method the *in vitro* drug permeation study was performed in two stages using the same locally fabricated diffusion cell. In the first stage PBS (pH 7.4) was used as the receptor medium and method as described above for skin permeation was carried out. UF-2 (2 mL) was applied to epidermal surface of rat skin. Samples were withdrawn through the sampling port of the diffusion cell at predetermined intervals over 10 hr and analyzed. The receptor phase was immediately replenished with equal volume of fresh buffer. At the end of 10 hr the donor compartment was washed five times with warm receptor fluid. The second stage used 50% v/v ethanol as the receptor solution for a further period of 12 hr and performed without any donor phase. During this stage an ethanolic receptor will diffuse into the skin disrupting the vesicular structure of any UF-2 that may have penetrated and deposited in the tissue and thus releasing both UF bound and free dexamethasone for collection by receptor fluid (Figure 6). Use of 50% ethanol as receptor fluid can slightly reduce the barrier nature of stratum corneum; hence, the second stage was performed after removal of the donor to avoid any excess permeation due to penetration enhancing activity of ethanol [24].

### 2.6. *Ex Vivo* Skin Permeation

For the evaluation of mechanism of better skin permeation ability of optimized oleic acid vesicles, skin interaction study was carried out. The vesicle-skin interaction of optimized ufasomal formulation (UF-2) was evaluated by scanning electron microscopy technique and FT-IR.

#### 2.6.1. Scanning Electron Microscopy

**Figure 7:**
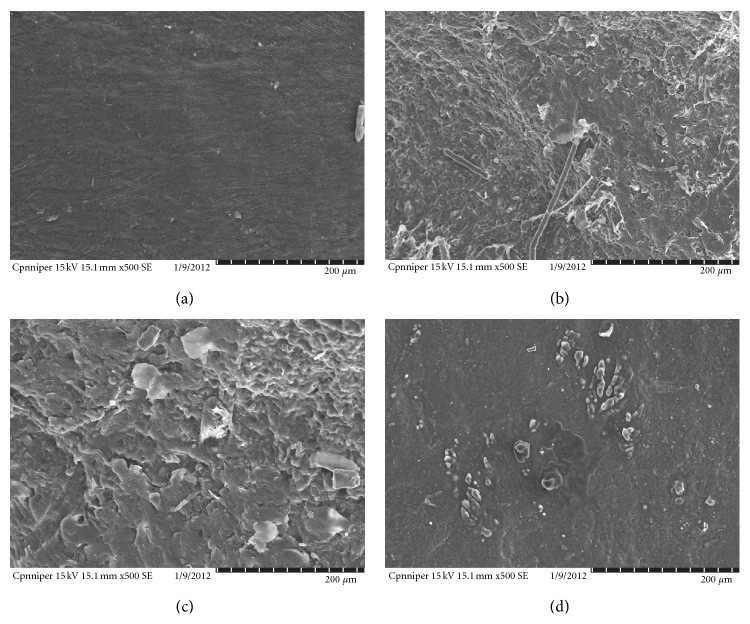
SEM photomicrograph (a) control, (b) liposomal formulation, (c) plain gel, and (d) UF treated rat skin.

SEM studies using rat skin was conducted in order to explain the effect of oleic acid formulation on the surface morphology of skin.  Figure 7 shows the SEM photomicrograph of rat skin treated with PBS (pH 7.4) acting as control, plain ufasomal gel, liposomal formulation, and plain gel. In rat skin incubated with PBS there was absence of intracellular vesicular structures in stratum corneum. However ufasomes were visualized on the surface of stratum corneum. The vesicular suspension formed networks and stacks of lipid bilayers at the interface of the stratum corneum (Figure 7). Intracellular vesicular structures were observed in superficial layers of the stratum corneum and their appearance might be explained by desquamating corneocytes with a leaky membrane, through which oleic acid vesicles penetrate. These vesicular suspensions are more flexible and can easily pass through skin pores. Skin treated with UF complex have rough surface with pore formation. Rough surface is probably due to the lipid perturbation effect of oleic acid.

#### 2.6.2. Fourier Transform Infrared Spectroscopy (FTIR)

**Figure 8:**
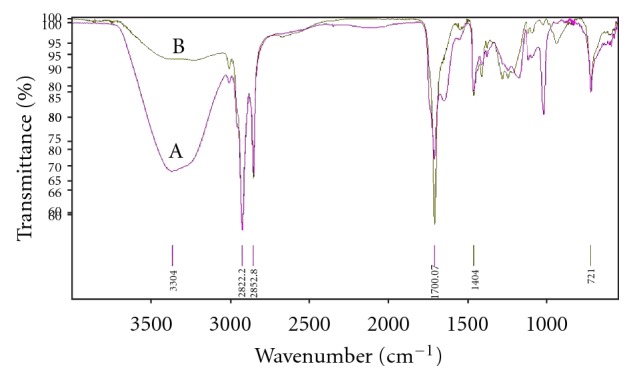
FTIR spectra (A) rat skin without treatment, (B) treatment with optimized formulation after 24 hr.

After skin permeation study of 24 hr SC (stratum corneum) was cut into small circular discs. The FT-IR spectra of dexamethasone, rat skin, and dummy formulation were measured on Perkin Elmer, USA (Model 1600) (Figure 8). The data was obtained in the range of 400–4000 cm^−1^ for each sample. Analyses were performed at room temperature.

### 2.7. *In-Vivo* Studies 

#### 2.7.1. Inhibition of Acute Edema Produced by Injection of Carrageenin

The study was carried out on male Wistar rats (150–200 g, *n* = 15) according to protocol number [IAEC/CCP/12/PR-008]. Thirty minutes before the intraperitoneal injection of each compound, the basal volume of the hind paws was measured by means of a mercury plethysmometer (Ugo Basile). Afterwards, the gel formulations were applied topically on the rat skin at the dose of 1 mg/Kg: (a) plain gel; (b) ufasomal gel formulation −0.5% w/w (1 square cm area); (c) marketed formulation topically −0.5% w/w. Thirty minutes after the treatment, carrageenin (0.05 mL of 1% suspension in saline) was injected intraplantarly into the right hind paw of each rat to induce inflammation and 0.05 mL of saline into the contralateral paw. Paw volumes up to the ankle joints were measured before and at hourly intervals for 6 h following carrageenin administration. The basal volume of each rat paw was taken as 100% and variations from this volume were given as percent difference [25].

### 2.8. Storage Stability Studies

The purpose of the study is to determine the effect of storage at different temperature conditions on stability of ufasomes. Physical stability studies were conducted by monitoring the change in mean vesicle size and the leakage of encapsulated drug from ufasomal formulations at different time intervals up to 30 days. The formulations were placed in tightly sealed vials flushed with nitrogen gas and stored at 4 ± 1°C and ambient temperature (28 ± 1°C) [26].

### 2.9. Statistical Analysis

All the results are expressed as mean ± standard deviation (SD). Statistical analysis was carried out employing the Student's *t*-test using the software PRISM (Graphpad). A value of *P* < 0.05 was considered statistically significant.

## 3. Results and Discussion

### 3.1. Fatty Acid Vesicle Characterization

Film hydration method was used for preparation of multilamellar oleic acid vesicles by varying ratios of oleic acid to dexamethasone followed by hydration at ambient temperature for 1 h. The thickness and uniformity of the film depends on speed of rotation. Uniform thin lipid film was formed at the rotation speed of 120 rpm while lower and higher rate of rotation resulted in detectable nonvesicular aggregated artifacts. The formed ufasomal formulations were further characterized for size, entrapment efficiency, and zeta potential studies (Table 1).

#### 3.1.1. Vesicle Shape

The photomicrographs showed that oleic acid vesicles formed were spherical in nature (Figure 1(a)). Further, to detect the multilamellarity of vesicles, TEM study was conducted (Figure 1(b)).

#### 3.1.2. Vesicle Size and Size Distribution

The size of the ufasomal formulations before sonication was observed in the range of 4 to 5 *μ*m while after sonication the vesicle size was in the range of 100–200 nm. For better skin permeation the vesicle size must be in the range of 100–200 nm. Hence, the result indicates that vesicle size was dependent on the composition of lipid bilayer. As the concentration of fatty acid in the oleic acid vesicular formulation was increased, there was an increase in the vesicle size to optimum value. However, reduction in vesicle size was observed when oleic acid concentration was increased above 15% w/w. This was due to the formation of micellar structure instead of vesicles, which has smaller size than vesicles [27, 28]. The result of the vesicle size measurement was well correlated with the report of [29] that vesicle size decreased as surfactant concentration was increased (Table 1).

The mean average diameter of ufasomes and traditional liposomes vesicles was 100 nm and was transparent colloidal dispersions (Figure 2) (Table 1). The ufasomes were homogenized by extrusion through a 100 and 200 nm polycarbonate membrane filters which result in the formation of monodispersed particles because of the low polydispersity index, that is, less than 1 (Table 1). The size of ufasomal formulation should be less than 50 nm so that it can be used for topical purpose. To support above facts we optimized and reported two parameters which can be related to stability of vesicular formulation. Sonication time of ufasomal formulation in size range of 100 nm was optimized at 40 W output frequency at various time intervals of 5, 10, 15, 20, and 25 min and the size was determined using DLS method. The sonication time required for preparing ufasomal formulation of size 100 nm was 12 min.

#### 3.1.3. Entrapment Efficiency

The studies carried out showed no significant difference in the entrapment efficiency; however the mean entrapment efficiency of the fatty acid vesicles was found to be increased on increasing the molar quantity of dexamethasone up to 8 : 2 oleic acid-to-dexamethasone ratio (65.2 ± 3.8%). Beyond this ratio no further increase in drug entrapment was recorded. The size of fatty acid vesicles was obtained in the range of 400 nm to 1 *μ*m. The oleic acid vesicular formulations were mostly poly disperse in nature. At 8 : 2 oleic acid-to-dexamethasone ratios the dispersity was recorded to be 0.534 ± 0.026. The photomicrographs showed that oleic acid vesicles formed were spherical in nature (Figure 2(a)). Further, to detect the multilamellarity of vesicles, TEM study was conducted (Figure 2(b)).

#### 3.1.4. Turbidity Measurements

Transformation of fatty acid vesicles to mixed micelles is concentration and pH dependent process and was governed mainly by progressive formation of mixed micelles within the bilayer. To support the above fact, turbidity measurements were performed. The results of turbidity measurement studies (Table 1) support the fact that micelles were formed at higher concentration of fatty acid. Turbidity of UF increased with increasing surfactant concentration. This can be observed due to fact that at low concentration of surfactant partition coefficient favors the lipid phase and caused expansion of lipid bilayer resulting in increased turbidity of vesicle dispersion. At the same time, surfactant also causes fluidization of bilayer, which is also responsible for increasing the turbidity. After an optimum concentration, conversion of lipid vesicles into mixed micelles begins which have negligible turbidity [30].

### 3.2. Preparation of Vesicles

The fatty acid vesicles were evaluated to assess their efficacy in delivering the bioactives to and through stratum corneum of the skin. The drug dexamethasone was used as a model drug. The major consideration in the formulation of fatty acid vesicles is pH of the formulation; this being a critical factor that controls the degree of ionization of fatty acid [14, 31] and is hence responsible for the formation of vesicle. The fatty acid (oleic acid) assembled into vesicles only if pH equals the pKa of the acid (8.5). At this pH, *~*50% of the carboxylic acid is ionized and transforms into ionized amphiphile(s) with a tendency to form vesicles/aggregates. The acid is present as ionic RCOO– as well as neutral RCOOH species. In such conditions each ionized group is stabilized through a strong hydrogen bond formed with the neutral molecules. The negative charge present on the ionized carboxylic group is shared between two adjacent fatty acid molecules, that is, ionized and unionized, and thus results in the formation of typical dimmers. The hydrophobic hydrocarbon chain of so formed dimmers protects itself from the aqueous compartment and thus orients to form an enclosed bilayer structure that minimizes the interaction between the hydrocarbon chain and water. The ratio of protonated and deprotonated group seems also critical in the process of vesiculation. This is possible only if the concentration of the fatty acid in aqueous dispersion exceeds the critical vesicle concentration (CVC), which is reportedly 80 mM for oleic acid [32]. The stability of the vesicles is attributed to the strong hydrogen bond-based interactions between the protonated and deprotonated groups, namely, RCOO–HOOCR [13, 33–35], the effect of drug : oleic acid ration on the encapsulation of the dexamethasone was studied. It was seen that the drug bearing capacity of the oleic acid vesicles depends upon the molar ratio of oleic acid and dexamethasone. The entrapment efficiency increased up to oleic acid : drug molar ratio 8 : 2; beyond this ratio further increase in the amount of drug reduced the degree of drug encapsulation. This may be ascribed to the drug saturation in the bilayer domain. Further addition of drug could have destabilized the vesicle membrane leading to the leakage of content. Since the 8 : 2 vesicles showed the best physicochemical characteristics (high homogeneity, high entrapment efficiency), the respective formulation was selected for further studies.

### 3.3. *In Vitro* Performance of Ufasomes through Cellophane Membrane

Different ufasomal formulations were subjected to *in vitro* drug release studies using cellophane membrane. For the optimization of drug concentration that could be incorporated into the vesicles, these ufasomal formulations were subjected to *in vitro* drug release through cellophane membrane and the % amount of drug permeated was calculated. Values of % amount of dexamethasone permeated across cellophane membrane are shown in Figure 3. By comparing the release from various oleic acid vesicles based formulations (prepared using different oleic acid and dexamethasone molar ratios), it was deduced that there was no considerable difference in the release in terms of kinetic pattern, although drug release exhibited a concentration-dependent behavior. These results are in agreement with the release kinetic reported by other research workers who documented that drug release from vesicles is affected by diffusion [36, 37].

The release rate of dexamethasone from ufasomes was slow, controlled and uniform than drug solution. After 4 h 98.5% drug was released from drug solution. In comparison at 8 hr, about 34.3% and, in 24 hr, 38.9% of dexamethasone were released from the ufasomal formulation (UF-2).

### 3.4. pH-Dependent Stability

The pH-dependent stability behavior substantiates that drug diffusion across the skin may increase with a decrease in the pH of the vesicles dispersion. Thus, the increased diffusion of drug from the vesicles at low pH may have resulted due to decreased stability of the vesicles at lower pH. This further suggests that vesicles tend to fuse when they are exposed to low pH. This particularly holds for the pH that is lower than physiological pH.

The study demonstrated the pH-dependent nature of the oleic acid vesicles. It also provided useful information on topical drug delivery potential and characteristics of oleic acid vesicles since the pH of skin is 7.2. It was observed that the release from vesicles is highly pH-dependent and on lowering the pH from 8.5 to 5.5, only 30% of the drug remained in vesicles to be released after 8 h of incubation in buffer of pH 5.5 as compared to residual drug estimated, that is, 71% at pH 8.5 (Figure 4). The differences in drug diffusion recorded at pH 8.5 and 7.4 were not significant (*P* > 0.01). Therefore, further studies were continued by adjusting the pH of vesicles suspension to 7.4, since higher pH values may cause skin irritation and may not be acceptable for topical application.

Simultaneously, morphological changes in vesicles size and shape were also observed with changing pH. The results displayed an increase in the size of the vesicles at low pH values (Figure 5).

### 3.5. Skin Permeation Study

The skin permeation study was conducted on optimized formulation prepared at 8 : 2 fatty acid : drug ratio (pH 7.4) (highest entrapment efficiency and more uniform sized vesicles). To normalize the effect of pH on skin permeation, the plain pH of drug gel was also adjusted to pH 7.4. A significant increase in the skin permeation of dexamethasone was recorded from oleic acid vesicle dispersion in comparison to plain gel (Figure 6). The amount of dexamethasone permeated from the plain Carbopol gel was 18.72%. The drug penetration following application of an equivalent amount of drug in vesicular dispersion was significantly high, that is, 34.75%.

The permeation parameters were calculated by plotting a curve between cumulative amounts of drug permeated per unit area (*μ*g/cm^2^) versus time. The flux was obtained from the slope of the linear portion of the graph. The transdermal permeation rate constants obtained were higher for oleic acid vesicle dispersions (18 ± 1.48 *μ*g/h/cm^2^) than the plain drug gel (3.8 ± 0.8 *μ*g/h/cm^2^) (Table 2).

### 3.6. Scanning Electron Microscopy (SEM)

SEM studies using rat skin were also conducted in order to explain the effect of fatty acid vesicles on the surface morphology of skin. After 24 hr application of optimized ufasomal formulation (UF-2) on to rat skin SEM photomicrographs were taken. Skin incubated with PBS acts as control (Figure 7). There was absence of intracellular vesicular structures in stratum corneum incubated with PBS. However ufasomes were visualized on the surface of stratum corneum. The vesicular suspension formed networks and stacks of lipid bilayers at the interface of the stratum corneum. Intracellular vesicular structures were observed in superficial layers of the stratum corneum and their appearance might be explained by desquamating corneocytes with a leaky membrane, through which liposomes penetrate. These vesicular suspensions are more flexible and can easily pass through skin pores.

In comparison to control skin, vesicle treated skin was more smooth and some vesicular structures were present on the surface. The morphology of cell was changed and some partial disappearance of intercellular lipid was observed. The increase in the intralamellar distance of SC lipids was also observed.

### 3.7. Fourier Transform Infrared Spectroscopy (FTIR)

Figure 8 shows the FTIR spectra of untreated skin (control) and skin treated with optimized formulation (UF-2). In pure dexamethasone spectra, the characteristic absorption bands at 3390 and 1268 cm^−1^ were due to the stretching vibration of O–H and C–F bonds, respectively; the stretching vibration at 1706, 1662, and 1621 cm^−1^ were due to –C=O and double bond framework conjugated to –C=O bonds [38].

FT-IR spectrum of untreated SC (control) showed various peaks due to molecular vibration of proteins and lipids present in SC (Figure 8(a)). The absorbtion bands in the wave number of 3000–2700 cm^−1^ were seen in untreated SC. These absorption bands were due to the C–H stretching of the alkyl groups present in both proteins and lipids. The bands at 2922.26 cm^−1^ and 2852.98 cm^−1^ were due to the asymmetric -CH_2_ and symmetric -CH_2_ vibrations of long chain hydrocarbons of lipids, respectively. The bands at 2955 cm^−1^ and 2870 cm^−1^ were due to the asymmetric and symmetric CH_3_ vibrations, respectively. These narrow bands were attributed to the long alkyl chains of fatty acids, ceramides, and cholesterol which are the major components of the SC lipids.

The sharp peak at 1708 cm^−1^ was due to the -C=O stretching vibrations of SC proteins. There was clear difference in the FTIR spectra of untreated and treated SC with prominent decrease in asymmetric and symmetric CH- stretching of peak height and area (Figure 8(b)).

The rate limiting step for topical drug delivery is lipophilic part of SC in which lipids (ceramides) are tightly packed as bilayers due to the high degree of hydrogen bonding. The amide I group of ceramide is hydrogen bonded to amide II group of another ceramide and forming a tight network of hydrogen bonding at the head of ceramides. This hydrogen bonding makes stability and strength to lipid bilayers and thus imparts barrier property to SC. When skin was treated with ufasomal formulation (UF-2), ceramides got loosened because of competitive hydrogen bonding leading to breaking of hydrogen bond networks at the head of ceramides due to penetration of ufasomes into the lipid bilayers of SC. Treatment with ufasomes resulted in either double or single peak at 1708 cm^−1^ (Figure 8(b)) which suggested breaking of hydrogen bonds by ufasomes.

### 3.8. Anti-Inflammatory Activity

**Figure 9:**
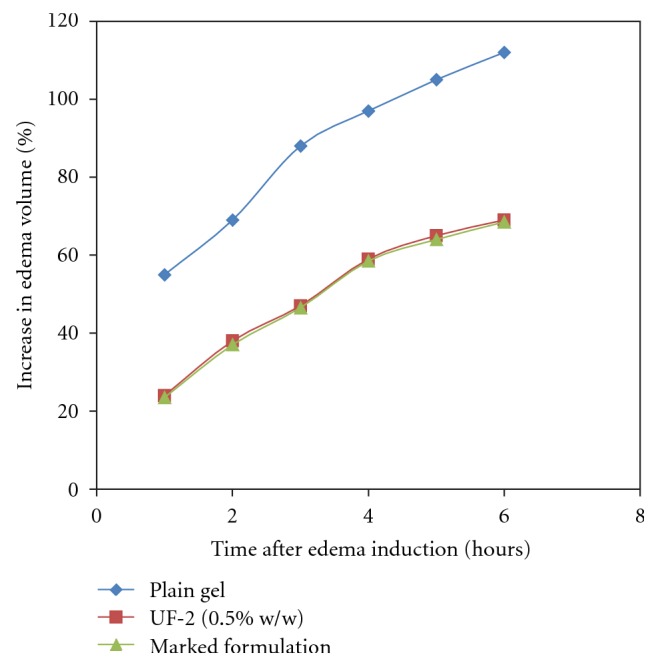
Increase in edema volume (%) in anti-inflammatory activity evaluation of ufasomes containing dexamethasone (0.5% w/w), plain gel “and” marketed formulation using the method of acute edema inhibition produced by carrageenin injection (5 rats per group) (ANOVA, F test).

Due to the best results observed in the physicochemical characterization, UF-2 formulation was chosen to undergo the pharmacological studies.  Figure 9 shows the increase in edema volume (%) using the method of acute edema inhibition produced by carrageenin injection, as a function of time. The evaluation of the anti-inflammatory activities was performed by the comparison of UF-2 with a dexamethasone commercial injection product (Decadron) used as reference. When dexamethasone was associated with fatty acid vesicles and its anti-inflammatory activity evaluated by inhibition, carrageenan edema a significant reduction of edema (*P* < 0.10) was measured in comparison to the commercial product.

### 3.9. Stability Studies

Stability of the product may be defined as the capability of a particular formulation to remain with the physical, chemical, therapeutic, and toxicological specifications. The optimized formulation (UF-2) was selected for stability study on the basis of its *in vitro *performance and stored in tightly closed glass vials at room temperature and in refrigerator (4 ± 2°C). Following parameters were evaluated at different time intervals (10, 20, and 30 days). The formulations were stored in 10ml glass vials at refrigeration temperature (4 ± 2°C) and room temperature for a period of one month. The samples were analyzed at predetermined time intervals visually and under optical microscope for the change in consistency and appearance of drug crystals.

#### 3.9.1. Physical Stability

The magnitude of drug retained within the vesicles ultimately governs the shelf life of the formulation. The fatty acid-based vesicles formulations when stored at ambient and refrigerated conditions have shown that the vesicles are stable at 4 ± 1°C. This is because the vesicles sizes at this temperature remain stable and unchanged; thus the leakage from the vesicles was minimal. The increase in size indicates intervesicular fusion. At ambient temperature (28°C), the phase transition temperature of oleic acid is exceeded; hence they tend to fuse [39, 40]. The drug leakage studies carried out also suggested better stability of fatty vesicles at refrigerated conditions.

## 4. Conclusion

 Based upon above results it can be concluded that encapsulation of drug in fatty acid vesicles can serve as potential carriers for the delivery of anti-inflammatory drug. Cost effectiveness, therapeutically viability, and sustained release behaviour along with drug retention in the deeper part of skin might be beneficial for the long-term effects of drugs. The oleic acid vesicles seemingly fuse with the skin and release the contents. They are seen to penetrate intact and to form drug depots in the skin. The fatty acid in addition may serve as a penetration enhancer, thus by avoiding the stratum corneum barrier potential they may lead to better permeation of the drug molecules.

## Figures and Tables

**Table 1:** Size and entrapment efficiency of the prepared oleic acid vesicles.

Formulation code	Oleic acid : drug	EE (%)	Particle size (nm)	PDI	Turbidity (500 N.T.U.)	Zeta potential (mV)
UF-1	9 : 1	39.6 ± 2.1	502 ± 18	0.321 ± 0.021	415 ± 10	−11.8 ± 0.9
UF-2	8 : 2	65.2 ± 3.8	631 ± 15	0.534 ± 0.026	387 ± 12	−6.8 ± 0.9
UF-3	7 : 3	53.4 ± 2.7	537 ± 16	0.284 ± 0.015	395 ± 8	−13.8 ± 0.9
UF-4	6 : 4	48.4 ± 2.5	525 ± 11	0.476 ± 0.029	421 ± 11	−7.8 ± 0.9
UF-5	5 : 5	44.5 ± 2.2	450 ± 13	0.438 ± 0.019	407 ± 10	−12.8 ± 0.4
LIPO CHL : PC	1 : 5	42.6 ± 2.4	474 ± 12	0.519 ± 0.022	392 ± 13	−2.8 ± 0.9

UF-1, 2, 3, 4, 5: ufasomal formulations, CHL: cholesterol, PC: phosphatidylcholine, EE: entrapment efficiency, PDI: polydispersity index, N.T.U.: nephelometric turbidity unit.

**Table 2:** Transdermal permeation parameters of different dexamethasone formulations across rat skin.

Permeation parameters	UF-2	Liposome	Plain drug gel
Jss^a^ (*μ*g/cm^2^/hr)	18 ± 1.48	8.72 ± 1.4	3.8 ± 0.8
LT^b^ (hrs)	2.5 ± 0.1	6.3 ± 0.7	8.2 ± 0.9
*P* ^d^ (cm/hr)	21.67	25.87	16.53
*R* ^2e^	0.792	0.852	0.773
De^f^ (*μ*g)	91.6 ± 1.7	38.5 ± 1.2	21.4 ± 1.6
ER^g^	4.74	2.29	—

Data represents mean ± SD (*n* = 3).

Jss^a^: transdermal flux; *R* ^2e^: correlation coefficient; LT^b^: lag time; De^f^: Amt. of drug deposited in skin; *P* ^d^: permeability coefficient; ER^g^: enhancement ratio.
